# Phenolic Content and Antioxidant Capacity in Algal Food Products

**DOI:** 10.3390/molecules20011118

**Published:** 2015-01-12

**Authors:** Ludmila Machu, Ladislava Misurcova, Jarmila Vavra Ambrozova, Jana Orsavova, Jiri Mlcek, Jiri Sochor, Tunde Jurikova

**Affiliations:** 1 Department of Food Analysis and Chemistry, Faculty of Technology, Tomas Bata University in Zlin, nam. T. G. Masaryka 5555, Zlin CZ-760 01, Czech Republic; E-Mails: lmachu@ft.utb.cz (L.M.); ambrozova@ft.utb.cz (J.V.A.); mlcek@ft.utb.cz (J.M.); 2 Language Centre, Faculty of Humanities, Tomas Bata University in Zlin, Mostní 5139, Zlin CZ-760 01, Czech Republic; E-Mail: orsavova@fhs.utb.cz; 3 Department of Viticulture and Enology, Faculty of Horticulture, Mendel University in Brno, Valticka 337, Lednice CZ-691 44, Czech Republic E-Mail: sochor.jirik@seznam.cz; 4 Department of Natural and Informatics Sciences, Faculty of Central European Studies, Constantine the Philosopher University in Nitra, Drazovska 4, Nitra SK-949 74, Slovakia; E-Mail: tjurikova@ukf.sk

**Keywords:** algae, phenols, Folin-Ciocalteu, antioxidant capacity of water soluble compounds, photochemiluminescence

## Abstract

The study objective was to investigate total phenolic content using Folin-Ciocalteu’s method, to assess nine phenols by HPLC, to determine antioxidant capacity of the water soluble compounds (ACW) by a photochemiluminescence method, and to calculate the correlation coefficients in commercial algal food products from brown (*Laminaria japonica*, *Eisenia bicyclis*, *Hizikia fusiformis*, *Undaria pinnatifida*) and red (*Porphyra tenera*, *Palmaria palmata*) seaweed, green freshwater algae (*Chlorella pyrenoidosa*), and cyanobacteria (*Spirulina platensis*). HPLC analysis showed that the most abundant phenolic compound was epicatechin. From spectrophotometry and ACW determination it was evident that brown seaweed *Eisenia bicyclis* was the sample with the highest phenolic and ACW values (193 mg·g^−1^ GAE; 7.53 µmol AA·g^−1^, respectively). A linear relationship existed between ACW and phenolic contents (*r* = 0.99). Some algal products seem to be promising functional foods rich in polyphenols.

## 1. Introduction

Algae, a group of marine or freshwater organisms, are traditionally used for direct consumption in Asian countries, and recently, their consumption as functional foods has also spread to Western countries. They are well known as an excellent source of biologically active compounds. Apart from high–quality proteins containing essential amino acids, dietary fiber, essential fatty acids, minerals, and vitamins, algae could also be a good source of phenolic compounds [1,2].

Reactive oxygen species (ROS) are chemically reactive molecules containing oxygen formed in mitochondria as a natural byproduct of energy production during the oxidative phosphorylation process. Ordinarily, the levels of free radicals in living organisms are controlled by a complex set of antioxidant defenses, which minimize oxidative damage to important biomolecules. Oxidative stress conditions are caused by endogenous oversized formation of ROS that exceeds the availability of antioxidants, and also by impact of external stressors. Excessive ROS can induce apoptosis and cause damage, especially to cellular proteins, polyunsaturated fatty acids, and DNA. Further, oxidative stress may be associated with nearly 200 diseases, such as cardiovascular diseases, cancer, atherosclerosis, hypertension, ischemia, diabetes mellitus, hyperoxaluria, neurodegenerative diseases (Alzheimer’s and Parkinson’s), rheumatoid arthritis, and aging, but it should not be considered the primary cause of these diseases [3].

Phenolic compounds in particular are considered as one of the most important classes of natural antioxidants. Their molecules are formed by one or more aromatic rings with one or more hydroxyl groups. Chemically, polyphenols can be divided into several classes, such as phenolic acids (hydroxybenzoic acids, hydroxycinnamic acids), flavonoids (flavones, flavonols, flavanones, flavanonols, flavanols, anthocyanins), isoflavonoids (isoflavones, coumestans), stilbenes, lignans, and phenolic polymers (proanthocyanidins—condensed tannins and hydrolysable tannins) [4]. Several thousand polyphenolic structures have been identified as secondary metabolites of plants. Polyphenols occur mainly in fruits and beverages, such as tea, wine, and coffee, and also in vegetables, leguminous plants, and cereals. Their concentrations in foods differ according to many factors (genetic, environmental, technologic, *etc.*); generally, phenolic acids account for one third of the total intake and flavonoids for the remaining two thirds, where the most abundant flavonoids in the diet are flavanols, anthocyanins, and their oxidation products [4]. Bioavailability within polyphenols differs considerably. As far as some compounds are concerned, it also depends on their form in their respective dietary sources [4]. Generally, their primary function in plants is as protection against ultraviolet radiation and pathogens [4]. Other roles include pigmentation, reproduction and growth of plants [5].

Phenolic compounds found in algae also include the phlorotannins found in brown algae and in lower amounts in some red algae. They are integral structural components of cell walls, but they have also been studied due to their other secondary ecological functions (e.g., protection from UV radiation, reproductive role in algal reproduction, protective mechanism against biotic factors), and also because of their therapeutic properties (anticancer, antioxidative, antibacterial, anti-allergic, anti-diabetes, anti-aging, anti-inflammatory and anti-HIV activities) [6,7].

Recently, polyphenolic compounds have become very common constituents of human diet and received an increasing interest from consumers and also from food manufacturers for many reasons, the health benefits, mentioned above, being the most significant [4].

Natural antioxidants are often multifunctional, therefore, it is proper to determine their antioxidant capacity. Because of their hydrophilic character, these compounds could influence the algal antioxidant capacity of water soluble compounds (ACW). This method developed by Popov and Lewin involves the photochemiluminescent (PCL) quantification of ascorbic acid in human plasma based on the ability of water-soluble antioxidants to scavenge O_2_ [8,9].

Although several studies have confirmed the attractive features of phenolic compounds and interesting antioxidant activity in fresh algae [10,11], data concerning phenolic content and ACW results in algal products are scarce, thus the purpose of this study was to investigate spectrophotometrically the total phenolic contents of algal food products and to determine selected phenolic compounds by HPLC in order to ascertain whether the phenolic contents of algal products are still relevant from the nutrition point of view, even after harvesting procedures, industrial treatments, and storage. Moreover, ACW values of the abovementioned samples were investigated by the PCL method and the relationships between obtained results were statistically evaluated by correlation coefficients.

## 2. Results and Discussion

Phenolic compounds are considered to be dominant contributors to the antioxidant activity and also possess many biological activities as mentioned in the Introduction section. Thus, the total phenolic content of nine algal food products was analyzed using the most appropriate extraction method considering the strenuousness of the laboratory procedure and cost-effectiveness. Moreover, nine phenolic compounds (*i.e*., gallic acid, 4-hydroxybenzoic acid, catechin hydrate, epicatechin, catechin gallate, epicatechin gallate, epigallocatechin, epigallocatechin gallate, pyrocatechol) were simultaneously determined by HPLC method to investigate the amounts of phenolic compounds in algal food products and further, to compare measured values to published data concerning fresh algae and other possible phenolic food sources. Then, ACW in algal extracts was determined by PCL method. In order to investigate whether there is a relationship between the total phenolic content and ACW, Pearson correlation and Spearman correlation coefficients were statistically evaluated. Also, Pearson correlation coefficients between either ACW or the total phenolic content and values of the selected phenolic compounds determined by HPLC were investigated.

### 2.1. Total Phenolic Content

The total amounts of phenolic compounds in the samples of edible algal products were determined spectrophotometrically after diverse kinds of extraction processes described in Section 3.3. The obtained results together with statistical evaluation can be seen in Table 1.

**Table 1:** Amounts (mg·g^−1^ GAE) of total phenolic content of edible algal products after various extraction processes: (1)—extraction by distilled water (80 °C for 10 min in water bath with constant shaking); (2)—extraction by methanol-water-acetic acid (30:69:1, v/v/v) (70 °C for 50 min in water bath with constant shaking); (3)—extraction by 80% methanol (70 °C for 1 h in water bath with constant shaking); (4)—extraction by 70% acetone (30 °C for 30 min in water bath with constant shaking); (5)—extraction by 100% methanol (lab temperature ≈ 23 °C for 24 h, constant shaking). Results are shown as mean ± SD (*n* = 4).

Algae	(1)	(2)	(3)	(4)	(5)
*Eisenia bicyclis*	192.6 ± 3.3 *^a^*	192.8 ± 0.8 *^a^*	143.2 ± 9.5 *^b^*	84.1 ± 0.3 *^c^*	9.5 ± 0.5 *^d^*
*Hizikia fusiformis*	34.5 ± 5.8 *^a^*	26.9 ± 0.1 *^b^*	9.5 ± 0.1 *^c^*	13.1 ± 0.1 *^c^*	6.0 ± 0.1 *^c^*
*Laminaria japonica*	8.7 ± 0.3 *^a^*	8.5 ± 0.3 *^a^*	14.9 ± 0.1 *^b^*	8.8 ± 0.1 *^a^*	0.7 ± 0.1 *^c^*
*Undaria pinnatifida* (W)	8.6 ± 0.4 *^a^*	3.7 ± 0.1 *^b^*	5.9 ± 0.1 *^c^*	5.7 ± 0.1 *^c^*	1.3 ± 0.1 *^d^*
*Undaria pinnatifida* (Wi)	8.0 ± 0.5 *^a^*	5.0 ± 0.1 *^b^*	6.5 ± 0.1 *^c^*	4.6 ± 0.1 *^b^*	8.4 ± 0.2 *^a^*
*Palmaria palmata*	31.8 ± 1.0 *^a^*	22.1 ± 0.7 *^b^*	26.5 ± 0.4 *^c^*	25.0 ± 0.1 *^d^*	10.7 ± 0.3 *^e^*
*Porphyra tenera*	18.2 ± 0.6 *^a^*	16.2 ± 0.4 *^b^*	15.1 ± 0.1 *^b^*	11.1 ± 0.1 *^c^*	4.7 ± 0.6 *^d^*
*Chlorella pyrenoidosa*	18.0 ± 0.2 *^a^*	13.2 ± 0.2 *^b^*	16.8 ± 0.1 *^a^^,c^*	15.5 ± 0.1 *^c^*	25.8 ± 1.7 *^d^*
*Spirulina platensis*	43.2 ± 1.0 *^a^*	17.0 ± 0.5 *^b^*	23.9 ± 0.1 *^c^*	18.4 ± 0.1 *^d^*	24.4 ± 0.2 *^c^*

^a–e^ values in the same line sharing a common letter are not significantly different at *p* < 0.05.

Generally, extraction using distilled water (extraction/**1**/) seemed to be the most effective procedure for almost all analyzed algal samples, except for the brown seaweed *Laminaria japonica* and the green freshwater alga *Chlorella pyrenoidosa*, where the extraction by 80% methanol (extraction/**3**/) and 100% methanol (extraction/**5**/), respectively, were the most efficient methods. In contrast, the last mentioned extraction process (extraction/**5**/) showed the lowest ability to extract phenolic compounds from the majority of algal products with a few exceptions, such as the brown seaweed *Undaria pinnatifida* (Wi) and the green freshwater alga *Chlorella pyrenoidosa*. Surprisingly, for these samples, this type of extraction was among the most effective ones.

Specifically, the highest and simultaneously multiply exceeding phenolic content was determined in the brown seaweed *Eisenia bicyclis* (193 mg·g^−1^ GAE). Quite high values were also ascertained in the cyanobacterium *Spirulina platensis* (43 mg·g^−1^ GAE). On the other hand, brown seaweed products from *Undaria pinnatifida* (W, Wi) showed absolutely the lowest values among all analyzed samples.

From the statistical point of view, one-way ANOVA revealed significant differences among the extraction processes of all algal samples; however, pairwise comparison occasionally disclosed similar efficiency of phenolic compound extraction methods.

Different algal products provided diverse total phenolic contents due to many influencing factors, such as algal species, geographical origin or the area of cultivation, seasonal, physiological, and environmental variations [12]. Type and conditions during the extraction also have a very decisive influence on the total phenolic content which is evident from Table 1 and from many other studies as well. For instance, the effects of ethanol, methanol, aqueous methanol and water extracts on phenolic contents and antioxidant capacity in brown alga were examined; the aqueous extract showed both the highest antioxidant capacity and highest phenolic contents, which is in accordance with our findings [13].

When comparing the brown seaweed *Eisenia bicyclis*, the sample with the highest content of total phenolic compounds, to the values determined in other possible sources of phenolic compounds, hardly any values exceeded measured data. For example, total phenolic content in 223 medicinal plants ranged up to 101.3 mg·g^−1^ GAE dry weight [14], in 62 fruits it varied up to 5.9 mg·g^−1^ GAE [15], and in 56 vegetables the highest value was 23.3 mg·g^−1^ GAE [16]. Just aqueous plant extracts made from industrial interesting plants surpassed the measured values (up to 397.0 mg·g^−1^ GAE). However, almost all of the phenolic rich extracts were made from parts of the plant which are not usually consumed in a raw form, *i.e.* bark and wood [17]. Well known sources of phenolic compounds, *i.e*., cocoa, red wine, and green tea constitute extraordinary exceptions; their phenolic contents of 611, 340 and 165 mg·g^−1^ GAE, respectively, surpassed the values, and only black tea (124 mg·g^−1^ GAE) had values comparable to the highest measured data [18]. According to research papers dealing with phenolic contents in fresh algae, the obtained algal product results cannot be properly compared because of different extraction conditions used; just for illustration—ethanol extract of *Eisenia bicyclis* contained 319 mg·g^−1^ GAE [19], aqueous extract of *Hizikia fusiformis* 4.1 mg·g^−1^ GAE [20], methanol–chloroform extract of *Laminaria japonica* 0.3 mg·g^−1^ GAE [21], aqueous extract of *Undaria pinnatifida* 3.8 mg·g^−1^ GAE [20], ethanol extract of *Palmaria palmata* 10.3 mg·g^−1^ GAE [22], aqueous extract of *Porphyra tenera* 10.1 mg·g^−1^ GAE [23], hexane, ethyl acetate and water fraction of *Chlorella pyrenoidosa* together contained 10.5–17.2 mg·g^−1^ GAE [24], and *Spirulina platensis* 19.5 mg·g^−1^ GAE [25]. The differences between phenolic contents could also be caused by the characteristics of the samples because all mentioned studies were realized with fresh algal samples contrary to the processed algal products analyzed in this work.

### 2.2. Selected Phenolic Compounds Content

Phenolic acids have been associated with many aspects of food quality including color, flavor properties, and nutrition [9]. Gallic and 4-hydroxybenzoic acid are representative of the hydroxybenzoic acid class and they are commonly present in a bound form as a component of more complex structures, such as lignins and hydrolysable tannins [4]. Gallic acid may itself be found conjugated, or as its dimer (ellagic acid), trimer (tergallic acid) and tetramer (gallagic acid). However, the last two substances exist relatively rarely, but along gallic and ellagic acids they could be esterified to glucose in hydrolysable tannins and gallic acid could also be esterified to condensed tannins [26]. Flavonoids obviously occur in fruits, vegetables, and other plant foods and they have been associated with reducing the risk of major chronic diseases. An inverse relationship between flavonoid intake and reduced risk of lung cancer was reported [4].

All edible algal products were analyzed by HPLC in order to establish amounts of the selected phenolic compounds, namely, gallic acid, 4-hydroxybenzoic acid, and further flavanols—catechin, epicatechin, catechin gallate, epicatechin gallate, egigallocatechin, epigallocatechin gallate and pyrocatechol. Obtained results are presented in Table 2.

Tabled results showed that epicatechin, which was the most frequently appearing phenolic compound, occurred in seven of nine samples analyzed. On the contrary, each of epicatechin gallate and pyrocatechol were detected only in one sample, the red seaweed *Porphyra tenera* and the cyanobacterium *Spirulina platensis*, respectively. The absolute highest amount of analyzed phenolic compounds was observed in the case of catechin in the sample of the red seaweed *Porphyra tenera* (129 μg·g^−1^). *Porphyra tenera* was also the sample with the highest sum of analyzed selected phenolic compounds and moreover, the sample with the most abundant presence of selected phenolic compounds—seven of nine compounds. Just two selected phenolic compounds, and also the lowest sum of phenolic compounds, were determined in the brown seaweed *Undaria pinnatifida* (W) and the red seaweed *Palmaria palmata*. The brown seaweed *Eisenia bicyclis*, previously (Section 2.1) evaluated as the sample with the highest total phenolic content, showed average results in comparison with other algal products.

**Table 2:** Amounts (μg·g^−1^ sample) of selected phenolic compounds (GA—gallic acid; HBA—4-hydroxybenzoic acid; C—catechin hydrate; EC—epicatechin; CG—catechin gallate; ECG—epicatechin gallate; EGC—epigallocatechin; EGCG—epigallocatechin gallate; PC—pyrocatechol) in edible algal products (A—*Eisenia bicyclis*; H—*Hizikia fusiformis*; K—*Laminaria japonica*; W, Wi—*Undaria pinnatifida*; D—*Palmaria palmata*; N—*Porphyra tenera*; C—*Chlorella pyrenoidosa*; S—*Spirulina platensis*).

	GA	HBA	C	EC	CG	ECG	EGC	EGCG	PC
A	2.8 ± 0.1	-	-	3.2 ± 0.3	2.9 ± 1.0	-	-	+	-
H	14.1 ± 0.5	-	-	8.2 ± 0.1	-	-	-	+	-
K	-	-	-	3.1 ± 0.1	-	-	4.0 ± 0.2	+	-
W	-	1.9 ± 0.1	-	-	-	-	4.8 ± 0.1	-	-
Wi	-	8.1 ± 0.1	-	6.3 ± 0.5	2.0 ± 0.2	-	21.4 ± 0.1	7.5 ± 0.1	-
D	-	5.8 ± 0.1	-	+	-	-	-	-	-
N	3.5 ± 0.1	1.6 ± 0.1	128.8 ± 2.9	16.4 ± 0.7	-	+	16.0 ± 0.5	4.0 ± 0.1	-
C	5.0 ± 0.2	20.5 ± 0.1	-	-	-	-	20.2 ± 0.7	-	-
S	-	-	22.7 ± 2.3	27.5 ± 1.3	-	-	-	-	28.9 ± 0.6

Results are shown as mean ± SD (*n* = 4); + trace amount; - not detected.

The distribution of the analyzed phenolic compounds in algal food products is very diverse and as in the case of total phenolic content, the differences could be caused by many factors (algal species, origin, growth conditions, *etc.*). Generally, the comparison of obtained results with similar studies focused on HPLC analysis of phenolic compounds/catechins in fresh algal samples [27,28] showed that the measured data were significantly lower than reported values. The immense difference can be illustrated by example of the most occurred phenolic compound in these studies—epigallocatechin; its contents varied from 130 to 3860 μg·g^−1^ [27] or from 252.5 to 760.2 μg·g^−1^ [28] which is much higher than the investigated values (4.0–21.4 μg·g^−1^). On the other hand, contents of 4-hydroxybenzoic acid in *Porphyra tenera* and *Undaria pinnatifida* (690 and 211 ng·g^−1^, respectively) mentioned in [29] were lower than the investigated ones (1.6 and 1.9/8.1 μg·g^−1^, respectively). It is obvious that algal food products contain many other phenolic compounds than were analyzed. Therefore, they deserve more detailed studies.

### 2.3. Antioxidant Capacity of Water Soluble Compounds (ACW)

**Figure 1:**
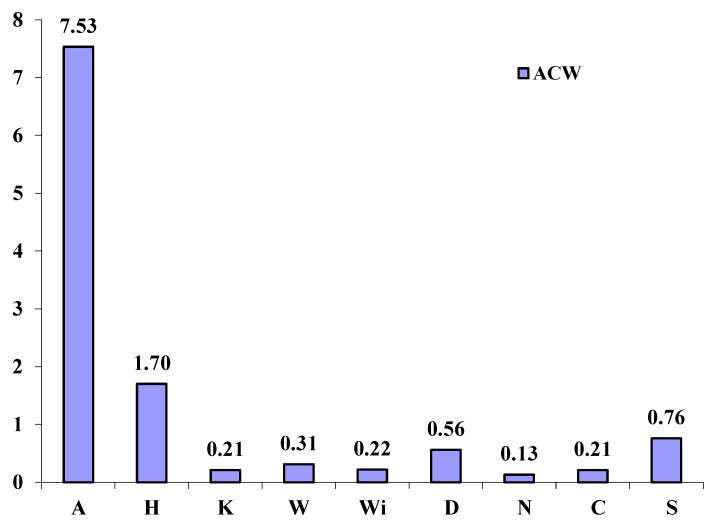
Values (µmol ascorbic acid·g^−1^ sample) of antioxidant capacity of the water soluble compounds (ACW) in edible algal products (A—*Eisenia bicyclis*; H—*Hizikia fusiformis*; K—*Laminaria japonica*; W, Wi—*Undaria pinnatifida*; D—*Palmaria palmata*; N—*Porphyra tenera*; C—*Chlorella pyrenoidosa*; S—*Spirulina platensis*).

ACW determined by PCL method was measured according to the procedure described in Section 3.6. Obtained values are shown in Figure 1.

As it can be seen, the highest value—7.53 μmol AA·g^−1^—was determined in the brown algal sample *Eisenia bicyclis*. This value exceeded other measured data several-fold. The brown algal product from *Hizikia fusiformis* (1.70 μmol AA·g^−1^), the product from the cyanobacterium *Spirulina platensis* (0.76 µmol AA·g^−1^), and the red algal product from *Palmaria palmata* (0.56 µmol AA·g^−1^) were the next samples with ACW values above 0.50 µmol AA·g^−1^.

When the total phenolic content data are compared with ACW values, noticeable similarities may be observed. For instance, algal food products with the highest total phenolic contents (*Eisenia bicyclis*, *Spirulina platensis*, *Hizikia fusiformis*, *Palmaria palmata*) were all in accordance with the algal samples rich in antioxidant capacity.

Obtained ACW results are very difficult to compare with other research studies dealing with ACW of algal food products because they are inaccessible. It is also impossible to compare measured data of antioxidant capacity of algal samples with the results acquired by another method (e.g., Trolox equivalent antioxidant capacity, or ferric reducing antioxidant power) due to the significant differences within the assays used [30]. Just for the illustration, ACW of some fruits rich in vitamin C were investigated by [31] and compared to baobab ACW values. This comparison resulted as follows: baobab (1.2–386.0 µmol AA·g^−1^ depending on the plant part), orange (17.0 µmol AA·g^−1^), bilberry (1.95 µmol AA·g^−1^), strawberry (1.72 µmol AA·g^−1^), and kiwi (0.73 µmol AA·g^−1^). A significant interesting source of ACW can be an ethanolic extract from the twigs of *Cinnamomum osmophloem* which was in the range from 91.3 to 3820.0 µmol AA·g^−1^, depending on specimens [32]. It may be stated that the investigated food algal products are not the samples with the highest ACW values; however, some of them have higher ACW than some fruits rich in vitamin C.

### 2.4. Statistical Correlations

**Figure 2:**
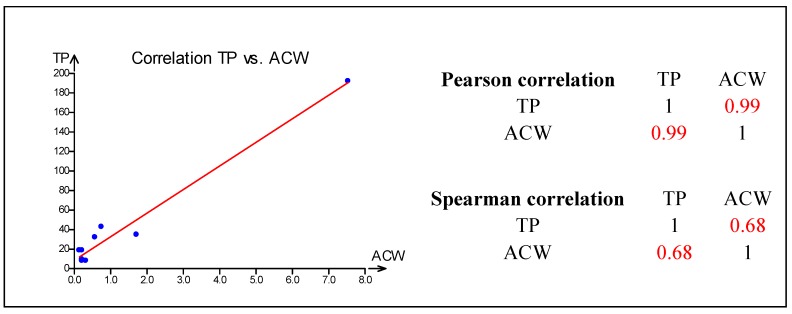
Graphic illustration of correlation between antioxidant capacities of the water soluble compounds (ACW) and total phenolic contents (TP) in algal food products together with matrixes of Pearson and Spearman correlation coefficients. The level of probability was set to *p* < 0.05.

**Figure 3:**
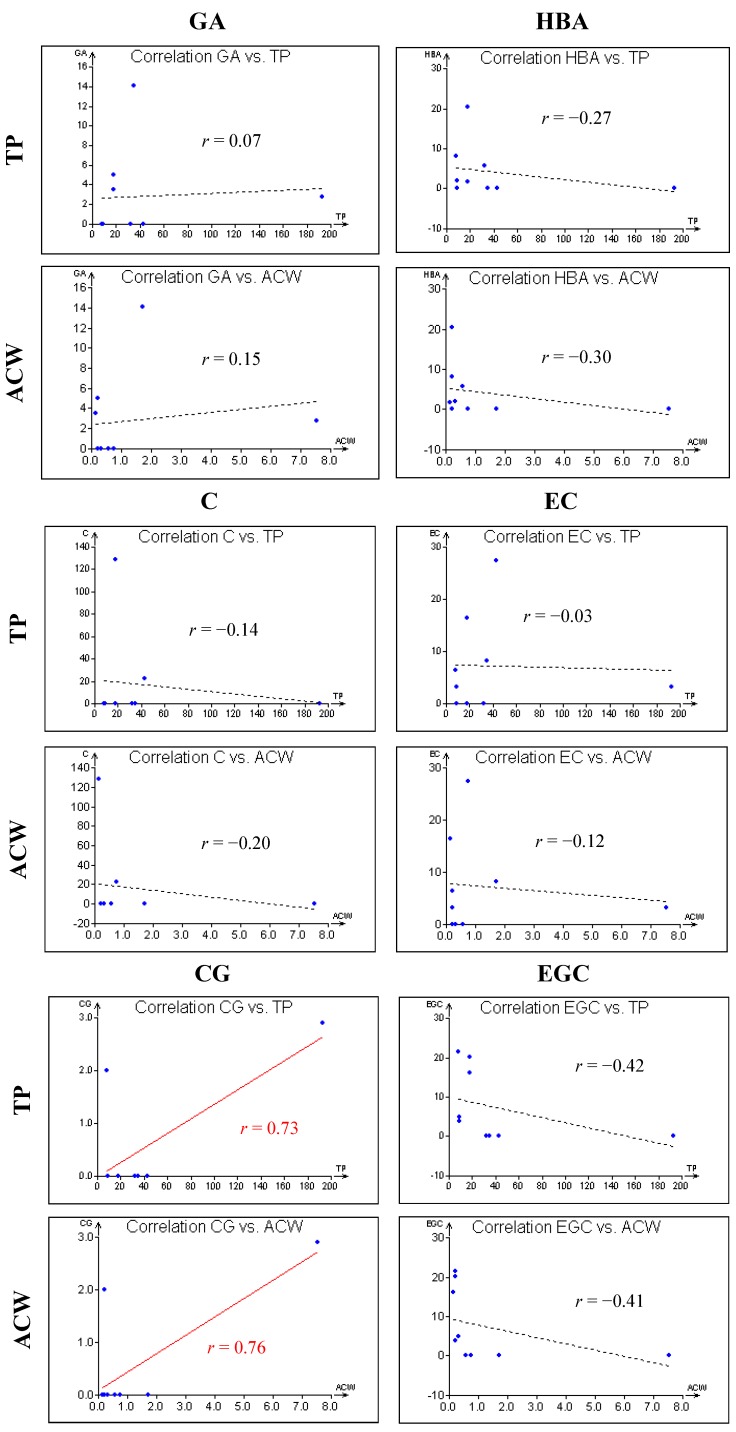

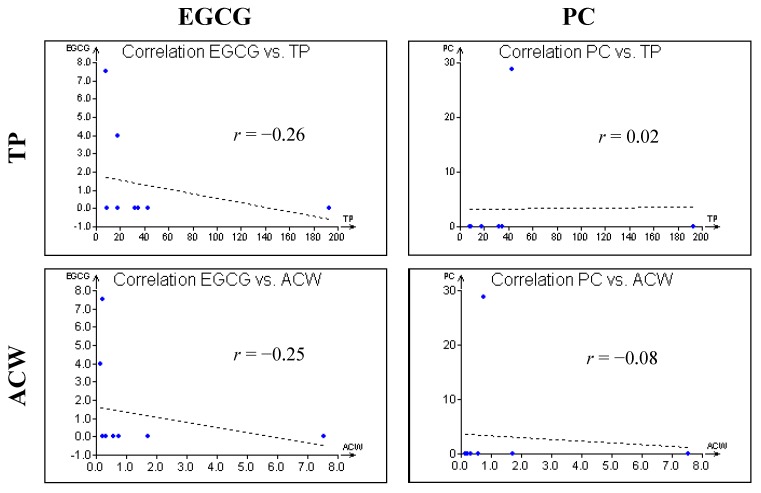
Graphic illustration of correlations between antioxidant capacities of either total phenolic contents (TP), or water soluble compounds (ACW) and amounts of selected phenolic compounds (GA—gallic acid; HBA—4-hydroxybenzoic acid; C—catechin hydrate; EC—epicatechin; CG—catechin gallate; EGC—epigallocatechin; EGCG—epigallocatechin gallate; PC—pyrocatechol) in algal food products together with values of their Pearson correlation coefficients (*r*). The level of probability was set to *p* < 0.05.

Because of some similarities in the obtained results, statistical correlations between ACW and the amount of total phenolic compounds have been investigated (see Figure 2). Moreover, correlation coefficients between either ACW, or the total phenolic compound contents and the amount of selected phenolic compounds (gallic acid, 4-hydroxybenzoic acid, catechin hydrate, epicatechin, catechin gallate, epigallocatechin, epigallocatechin gallate, pyrocatechol) were calculated (see Figure 3). Statistical evaluation by Pearson and Spearman correlation between the total phenolic content and ACW revealed quite high coefficients—0.99 and 0.68, respectively. So, it is evident that a close relationship exists between the total phenolic content and ACW of investigated algal food products. Also, in order to investigate whether there is a relationship between the investigated phenolic compounds determined by HPLC and either the total phenolic content, or ACW, statistical evaluation using Pearson correlation coefficients was performed as well.

As it can be seen in Figure 3, almost all evaluations show insignificant relationships. The only exception was a statistically significant correlation between catechin gallate and both ACW and total phenolic content with corresponding calculated Pearson correlation coefficients of 0.76 and 0.73, respectively. Although several studies confirmed the interesting medicinal properties of the investigated selected phenolic compounds, including antioxidant activity, obviously, there exist many factors influencing their display in algal food products. For example, in [33] it was reported that gallic acid in the concentrations of 10 and 20 mg·kg^−1^ showed significant protective effects on hepatic lipid peroxide metabolism, glycoprotein components, and lipids in diabetic rats. In [34] the authors revealed the hypoglycemic activity of 4-hydroxybenzoic acid in rats after the oral administration of 5 mg·kg^−1^. In [35] it was shown that catechin hydrate inhibits proliferation and mediates apoptosis of SiHa human cervical cancer cells. The *in vitro* cytotoxicity of catechin gallate was investigated by [36].

In [37] a protective effect of epicatechin and epicatechin gallate on lipid peroxidation in phospholipid bilayers was determined, while [38] revealed epigallocatechin gallate as a cancer preventive tea polyphenol in a mouse tissue. The impact of epigallocatechin gallate on a variety of human diseases, *i.e*., antioxidant, antiangiogenic or antitumor properties, was summarized by [39].

## 3. Experimental Section

### 3.1. Algal Samples

In the present study, algal products from brown seaweeds (*Laminaria japonica*, *Eisenia bicyclis*, *Hizikia fusiformis*, *Undaria pinnatifida*), red seaweeds (*Porphyra tenera*, *Palmaria palmata*), freshwater green algae (*Chlorella pyrenoidosa*) and cyanobacteria (*Spirulina platensis*) were analyzed. All the seaweed samples in the dried form together with freshwater algal samples in the form of tablets were purchased in health food stores. For additional information, see Table 3.

Algal samples were homogenized (Vorwerk Thermomix TM 31, Asbach, Germany) to a particle size of 1 mm and stored in plastic screw–cap bottles in dry, dark environment.

**Table 3:** Edible algal products characterization.

Species	Algal Product	Type	Country of Origin
*Eisenia bicyclis*	Arame	Brown	Japan
*Hizikia fusiformis*	Hijiki	Brown	Japan
*Laminaria japonica*	Kombu	Brown	Japan
*Undaria pinnatifida* (W)	Wakame	Brown	Japan
*Undaria pinnatifida* (Wi)	Wakame-instant	Brown	Japan
*Palmaria palmata*	Dulse flakes Bio	Red	USA
*Porphyra tenera*	Nori flakes	Red	Japan
*Chlorella pyrenoidosa*	Chlorella Tabs	Green	Taiwan
*Spirulina platensis*	Spirulina Bio	Cyanobacteria	India

### 3.2. Chemicals

All chemicals were of analytical grade. Methanol was from Fisher Scientific (Hampton, VA, USA). Acetonitrile was provided by Verkon (Praha, Czech Republic). Acetic acid was supplied by Penta (Praha, Czech Republic). Acetone together with l-ascorbic acid and sodium carbonate were provided by IPL (Uherský Brod, Czech Republic). Folin-Ciocalteu’s reagent was from Sigma-Aldrich (St. Louis, MO, USA). The ACW kit was a part of the PHOTOCHEM device (Analytik Jena AG, Jena, Germany). Standards of epicatechin, epicatechin gallate, epigallocatechin and epigallocatechin gallate were supplied by Extrasynthese (Genay Cedex, France). Catechin gallate, gallic acid, pyrocatechol and 4-hydroxybenzoic acid were provided by Sigma-Aldrich, and catechin hydrate was from Labicom (Olomouc, Czech Republic).

### 3.3. Phenolic Compounds Extraction

Five types of extraction mixtures were used. The composition of appropriate extraction mixtures was done according to the previous studies of research papers dealing with total phenolic content determination in various materials [23,28,40] in order to find out uncomplicated and cost-effective extractive procedures. Each homogenized algal sample (0.1 g) was accurately weighed into a screw-cap centrifuge tube and 10 mL of extraction mixture was added afterwards:

/**1**/ distilled water (80 °C for 10 min in water bath with constant shaking);
/**2**/ methanol–water–acetic acid (30:69:1, v/v/v) (70 °C for 50 min in water bath with constant shaking);
/**3**/ 80% methanol (70 °C for 1 h in water bath with constant shaking);
/**4**/ 70% acetone (30 °C for 30 min in water bath with constant shaking);
/**5**/ 100% methanol (23 °C for 24 h, constant shaking).

After the extraction, extracts were cooled to room temperature if necessary and centrifuged (EBA 20, Hettich, Kirchlengern, Germany) at 6000 rpm for 5 min. The supernatant was removed and immediately analyzed.

### 3.4. Total Phenolic Content

The total phenolic content of algal extracts was assessed according to the Folin-Ciocalteu method. Briefly, 1 mL of algal extract, 1 mL of Folin-Ciocalteu’s reagent and 5 mL of distilled water were mixed together. The solution was incubated for 5 min at the room temperature in the darkness. Then, 1 mL of 20% Na_2_CO_3_ was added. The solution was made up to 10 mL, mixed and incubated for 1 h at the room temperature in the darkness. The absorbance of algal sample was measured at 765 nm against a blank (corresponding extraction mixture was used instead of algal extract) on UV/VIS spectrometer Lambda 25 (PerkinElmer, Waltham, MA, USA). Gallic acid was used as a standard to construct the calibration curve (20, 40, 60, 80 and 100 mg·L^−1^). The total phenolic content of algal samples was expressed in mg·g^−1^ of gallic acid equivalent (GAE).

### 3.5. HPLC Analysis of Selected Phenolic Compounds

Chromatographic separation was accomplished on a C18 Kinetex (Phenomenex, Torrance, CA, USA) column (150 mm × 4.6 mm, i.d. 2.6 µm) using HPLC device UltiMate^®^ 3000 (Dionex, Sunnyvale, CA, USA) with a DAD detector. Conditions for the extraction and HPLC analysis were set according to [28]; however, partial modifications in order to optimize the results had to be performed.

Extraction /**2**/ (mentioned in Section 3.3) was evaluated as the most effective one for selected polyphenols extraction and, therefore, was used for HPLC analysis. After the extraction, obtained supernatant was filtered through 0.45 µm nylon LUT syringe filter (Labicom, Olomouc, Czech Republic) immediately before injecting into the chromatograph.

The HPLC analysis was performed using water–acetic acid (99:1, v/v) as mobile phase A and water-acetonitrile-acetic acid (67:32:1, v/v/v) as mobile phase B in the gradient mode (0–10 min: 90% A + 10% B, 10–16 min: 80% A + 20% B, 16–20 min: 60% A + 40% B, 20–25 min: 50% A + 50% B, 25–27 min: 60% A + 40% B, 27–35 min: 90% A + 10% B). The flow rate was 1 mL·min^−1^, the injection volume was 10 µL and the separation was performed at 23 °C. Chromatograms were registered at 275 nm. The identification of phenolic compounds was accomplished by comparison of their retention times with those of pure standards. Quantitative evaluation was performed with the standard addition method and consequent calculation.

### 3.6. Antioxidant Capacity of the Water Soluble Compounds (ACW)

Determination of ACW by photochemiluminescence (PCL) method using a PHOTOCHEM device (Analytik Jena AGwas realized according to the instruction manual provided by the manufacturer. This method is based on the blue luminescence of the detector substance luminol (5-amino-2,3-dihydro-1,4-phthalazinedione) caused by remaining radicals which were produced by optical excitation of a photosensitizer substance and partially eliminated from the sample by reaction with the antioxidants present in the sample [8,9].

For the analysis, extracts of algal food products created by extraction /**1**/ (mentioned in Section 3.3) were used. The antioxidant capacity of the sample was quantified by comparison with the standard (ascorbic acid was used as the standard to construct the calibration curve) and the results were given in µmol of ascorbic acid·g^−1^ sample (µmol AA·g^−1^).

### 3.7. Statistical and Data Analysis

All the experiments were performed four times (*n* = 4) and the results were expressed as the mean value ± standard deviation (SD). Values of total phenolic contents obtained by various extraction methods were statistically evaluated by means of one–way analysis of variance (ANOVA) and Scheffé’s pair wise comparison using QC Expert 3.3 statistical program (TriloByte Statistical Software, Pardubice, Czech Republic). Values of Pearson and Spearman correlation coefficients between antioxidant capacities of water-soluble substances and the amount of total phenolic compounds, and Pearson correlation coefficients between either antioxidant capacity of water-soluble substances, or total phenolic compound contents, and the amount of selected phenolic compounds (gallic acid, 4-hydroxybenzoic acid, catechin hydrate, epicatechin, catechin gallate, epigallocatechin, epigallocatechin gallate, pyrocatechol) were also calculated using the QC Expert 3.3 statistical program. The level of probability was set to *p* < 0.05.

## 4. Conclusions

Algae are a source of many biologically functional substances including phenolic compounds which deserve attention because of the many health benefits they provide. In this study, it was proven that algal products also have considerable amounts of total phenolic content which are comparable to phenolic content in black tea. However, it is needed to keep in mind that all algal polyphenols are responsible for the health benefits, not only single components. Thus, nutritionists recommend the intake of antioxidants through the consumption of whole foods, not only from individual dietary supplements. Interestingly, some algal food products showed higher ACW values than some fruits rich in vitamin C. Besides, statistical analysis proved a linear relationship between ACW values and total phenolic contents (*r* = 0.99) in the investigated algal food products. The algal product Arame from the brown alga *Eisenia bicyclis* should be emphasized because it seems to be a very promising functional food and also a phenolic source which deserves further detailed study.
